# Design and Simulation of Lamotrigine Intermittent Release from a Subcutaneous Implant with an Enzymatic Biosensor Based on Clinical Data

**DOI:** 10.3390/bios16060348

**Published:** 2026-06-21

**Authors:** Jovana Arsenović, Alisa Budak, Melinda Taši, Mladena Lalić-Popović, Nemanja Todorović, Maja Milanović, Nataša Milić, Nataša Milošević

**Affiliations:** 1 Department of Computing and Control Engineering, Faculty of Technical Sciences, University of Novi Sad, 21000 Novi Sad, Serbia; arsenovic.jovana@uns.ac.rs (J.A.); melindatasi03@gmail.com (M.T.); 2 Department of Pharmacy, Faculty of Medicine, University of Novi Sad, 21000 Novi Sad, Serbia; alisa.budak@gmail.com (A.B.); nemanja.todorovic@mf.uns.ac.rs (N.T.); maja.milanovic@mf.uns.ac.rs (M.M.); natasa.milic@mf.uns.ac.rs (N.M.); natasa.milosevic@mf.uns.ac.rs (N.M.)

**Keywords:** epilepsy, lamotrigine, implant, closed-loop system, biosensor

## Abstract

Epilepsy can be effectively controlled with appropriately selected antiepileptic drugs and carefully titrated dosage regimens. Although lamotrigine exhibits favorable pharmacokinetic properties following oral administration, fluctuations in plasma concentration may still occur due to interindividual variability, irregular dosing, and pharmacokinetic interactions. In this study, a subcutaneous implant capable of monitoring plasma lamotrigine levels and adjusting drug delivery accordingly was developed to maintain stable therapeutic concentrations. The proposed system combines intermittent drug release with continuous concentration monitoring using an enzymatic biosensor. A pharmacokinetic model based on first-order absorption and elimination kinetics was implemented in MATLAB/Simulink using clinical lamotrigine concentration data obtained from patients receiving chronic therapy. In the closed-loop configuration, biosensor measurements were used as feedback for a proportional–integral (PI) controller that adjusted the implant release rate in real time. System performance was evaluated using in silico simulations. The open-loop system produced rapid concentration peaks (Cmax ≈ 0.06 mmol/L) followed by a decline below the therapeutic threshold within approximately 80 min. In contrast, the closed-loop system achieved lower peak concentrations (Cmax ≈ 0.045 mmol/L) and maintained plasma concentrations within the therapeutic range of 0.02–0.03 mmol/L with reduced fluctuations. These findings support further investigation of biosensor-guided closed-loop lamotrigine delivery systems.

## 1. Introduction

Over 50 million people globally suffer from epilepsy, a chronic neuropathological condition characterized by a long-term predisposition to generate epileptic seizures, accompanied by neurobiological, cognitive, psychological and social consequences [1,2]. Low- and middle-income countries account for more than 80% of the total epilepsy burden [3]. The current classification of epilepsy is provided by the International League Against Epilepsy (ILAE), focusing on the clinical manifestation of the disease and defining three types: seizure type, epileptic type and epileptic syndrome [4]. According to estimates by the World Health Organization (WHO), after correct diagnosis and adequate therapy, up to 70% of people with epilepsy can live in remission [1,5].

Pharmacotherapy is indicated in individuals who have experienced at least two unprovoked epileptic seizures with an interval of more than 24 h, or one seizure with a high probability of recurrence due to cerebral malformations, and in the event that epileptiform changes are observed during electroencephalogram (EEG) recording [6,7]. The approach to treatment is individual and the selection of antiepileptic drugs is configured by many factors: seizure characteristics, presence of epileptic syndrome, age, gender, potential pregnancy, presence of comorbidities, polypharmacy, but also patient preference [6,7]. The initiation of chronic therapy is gradual and requires titration of the dose over several weeks until the desired antiepileptics blood levels are achieved, after which a maintenance regimen is initiated, which usually involves oral administration of a pharmaceutical dosage form (tablets, capsules, or suspensions) every 8 to 12 h [8]. The challenge in the pharmacotherapy of epilepsy is the entire spectrum of potential adverse reactions of the antiepileptics, such as cognitive and coordination disorders, sedation, effects on mood, emotions, and sleep, migraine, weight fluctuations, and changes in the skin and mucous membranes [9]. Thus, regular clinical and laboratory monitoring is required when more antiepileptic drugs are administered [10].

**Figure 1:**
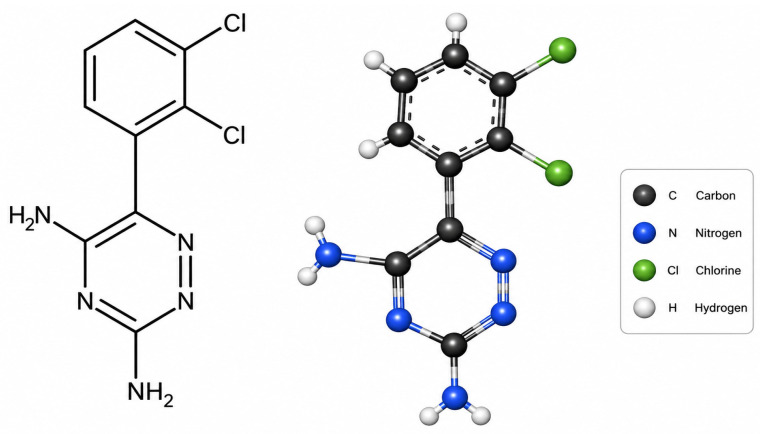
Structure of lamotrigine (LTG).

Lamotrigine (LTG) is an antiepileptic with a phenyltriazine structure (Figure 1) indicated for use as monotherapy or as part of combination therapy in focal and generalized epilepsies and clonic-tonic seizures, as well as a mood stabilizer in bipolar disorder [11]. It exerts its action by stabilizing the presynaptic membrane of neurons by inhibiting voltage-gated sodium and calcium channels [12], thereby modulating the release of excitatory neurotransmitters, primarily glutamate and aspartate [13].

After oral administration, the bioavailability of LTG is approximately 98%, the presence of food in the gastrointestinal tract does not interfere with absorption and it is not subject to the first-pass effect through the liver, and it reaches maximum blood concentrations within 2.8 ± 1.3 h [14,15]. The volume of distribution of LTG in patients with epilepsy is 1.25 to 1.47 L/kg, and more than half of the absorbed dose (55%) is bound to plasma proteins [15]. It is likely to cross the blood–brain barrier using organic cation transporters (OTC) [12], and is concentrated in brain tissue, reaching concentrations almost twice those of the free fraction of LTG in plasma (4.2 μg/g vs. 2.64 mg/L). During chronic administration, LTG exhibits first-order kinetics [14]. It is primarily inactivated in the liver by the enzyme UDP-glucuronyltransferase, to the major metabolites 2-N-glucuronide and 5-N-glucuronide and alternatively, to N-oxide and N-methyl, which can be detected in trace amounts [12]. Over 80% of total metabolites are excreted in the urine [12], and the elimination half-life (t1/2) of LTG ranges from 24.1 to 35 h [15].

Due to the numerous limitations associated with conventional antiepileptic pharmacotherapy, alternative therapeutic approaches that combine controlled drug delivery with continuous therapeutic monitoring should be considered. One potential solution involves the use of a subcutaneous implant capable of storing and intermittently releasing lamotrigine using an integrated pump system. In this context, two delivery strategies may be distinguished: an open-loop system with predefined drug release and a closed-loop system in which drug delivery is dynamically adjusted according to biosensor feedback. The incorporation of a biosensor therefore represents the key difference between these two approaches.

**Figure 2:**
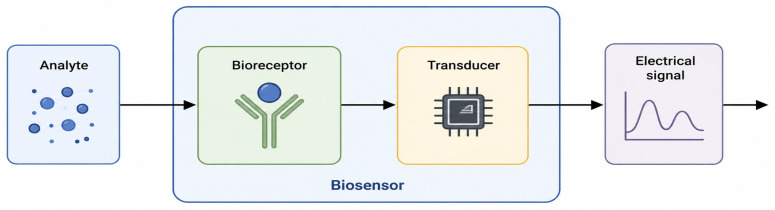
Block diagram of a typical biosensor [Image generated by AI based on existing images and schematics, with improvements made to visual quality, clarity, and presentation].

A biosensor (Figure 2) capable of detecting and quantifying biochemical analytes by converting concentration-dependent information into an electrical or optical signal was considered as a potential solution for continuous LTG monitoring [16]. A typical biosensor consists of two basic functional parts: a chemical (molecular) recognition system, the so-called bioreceptor, and a physicochemical transducer. A bioreceptor (enzyme, cell, aptamer, DNA, antibody, etc. [16]) is expected to translate the information from the biochemical domain, usually analyte concentration, into a chemical or physical signal with a defined sensitivity [17]. The transducer subsequently converts this biochemical response into an electrical signal suitable for further processing and feedback regulation [18]. In the proposed system, the biosensor was integrated into the closed-loop architecture in order to provide continuous information about plasma LTG concentrations and enable adaptive drug release.

Thus, the aim of this study was to develop and evaluate a subcutaneous implant-based system for controlled lamotrigine delivery and continuous therapeutic monitoring. The proposed system was designed to intermittently release LTG while simultaneously monitoring plasma drug concentrations using an enzymatic biosensor, enabling adaptive dose adjustment in order to maintain therapeutic concentrations within the desired range. Both open-loop and closed-loop control strategies were investigated, and the developed in silico models were evaluated using clinical plasma LTG concentration data obtained from patients receiving chronic therapy.

## 2. Materials and Methods

### 2.1. Clinical Data of LTG Plasma Concentrations

The system simulation was performed using previously published clinical data (Table 1) [19]. The data of 18 patients (55.56% female) treated with LTG only were observed for testing the biosensor. Ideal body weight (IBW) was calculated for each patient in kilograms as following [20]:

For men:

IBW = 50 + 0.91 × (H − 152.4)(1)

For women:

IBW = 45.5 + 0.91 × (H − 152.4),(2)

where H was the patient’s height given in cm.

**Table 1:** Anthropometric characteristics, LTG doses, and steady-state LTG plasma concentrations of the observed patients.

Parameter	Mean ± SD	Median
Age [year]	29.11 ± 11.40	27.5
Height [cm]	173.0 ± 8.83	172.5
Weight [kg]	68.72 ± 13.29	65.0
LTG dose [mg/day]	156.94 ± 74.16	137.5
LTG dose [mg/kg IBW/day]	2.44 ± 1.46	2.16
Steady-state LTG plasma concentration [μg/mL]	4.67 ± 3.66	3.86

Furthermore, the LTG dose prescribed to each patient was calculated per kg of the ideal body weight per day [mg/kg IBW/day]. The anthropometric characteristics, LTG doses, and steady-state LTG plasma concentrations of the observed patients are summarized in Table 1.

### 2.2. Design of LTG Monitoring and Control Systems

Two systems were designed in order to provide continuous LTG delivery and monitoring: an open-loop control system and a closed-loop system.

#### 2.2.1. Open-Loop Control Architecture

**Figure 3:**
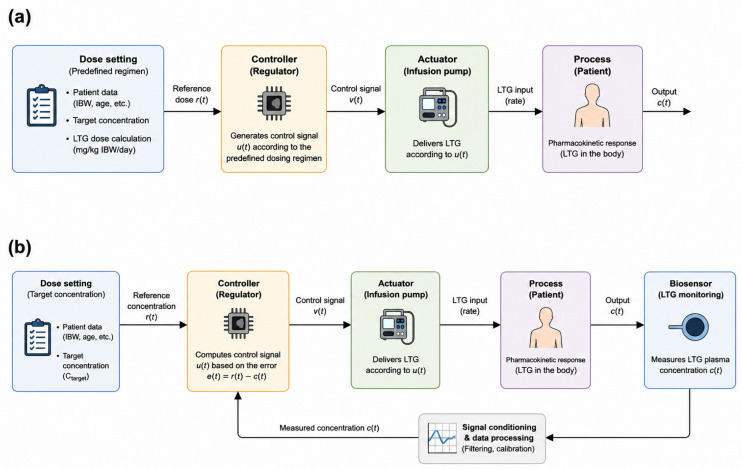
Overall architecture of the proposed LTG delivery and monitoring systems. Subfigures: (**a**) Open-loop control system for predefined LTG administration without feedback from measurements; (**b**) Closed-loop control system with biosensor-based feedback enabling real-time adjustment of LTG delivery according to the measured plasma concentration. [Image generated by AI based on existing images and schematics, with improvements made to visual quality, clarity, and presentation].

An open-loop control architecture was designed to provide predefined LTG delivery without feedback from the measured LTG plasma concentration (Figure 3a). In this configuration, the administered dose was determined in advance based on patient-related parameters and remained independent of real-time biosensor measurements. The controller generated the control signal corresponding to the LTG dosing regimen, while the process block represented the patient-specific pharmacokinetic response to the administered therapy [21].

#### 2.2.2. Design of a Closed-Loop System

A closed-loop control architecture was designed to enable continuous LTG monitoring and adaptive drug delivery based on real-time biosensor measurements (Figure 3b). In this configuration, the LTG plasma concentration measured by the biosensor was continuously fed back to the controller through the signal conditioning and data processing block. The controller compared the measured concentration with the predefined target concentration and generated the corresponding control signal for LTG administration. Consequently, the delivered dose could be dynamically adjusted according to the patient-specific pharmacokinetic response in order to maintain LTG concentrations within the desired therapeutic range [21].

#### 2.2.3. Biosensor Modeling

In addition to the system-level control architecture, a mathematical model of the enzyme biosensor was considered to describe the diffusion–reaction processes underlying LTG concentration measurements.

An enzyme biosensor operates according to the principle of a loop in which the substrate (S) is enzymatically converted into the first product (P1), followed by electrochemical conversion of the first product to the second product (P2), which is again enzymatically converted back into the first product. Thus, the level of the first product is detected [22].

**Figure 4:**
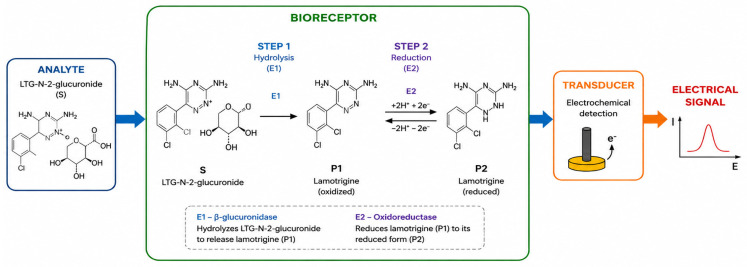
Proposed enzyme biosensor model for LTG monitoring. LTG-N-2-glucuronide (S) is enzymatically hydrolyzed by β-glucuronidase (E1) to produce lamotrigine (P1), which is subsequently reduced by an oxidoreductase enzyme (E2) to its reduced form (P2). The resulting electrochemical reaction is detected by the transducer and converted into an electrical signal proportional to LTG concentration. [Image generated by AI based on existing images and schematics, with improvements made to visual quality, clarity, and presentation].

The proposed biosensor model (Figure 4) catalyzes the hydrolysis of LTG-N-2-glucuronide (S) by the enzyme β-glucuronidase (E1), after which the released LTG (P1), under the action of enzymes from the oxidoreductase group (E2), is reduced (P2). This is followed by oxidation of the reduced form of LTG, which provides the electrons necessary for generating the electrochemical potential.

Since the enzymes that catalyze this process have not been specifically determined, the values of the required parameters were taken for β-glucuronidase that catalyzes the hydrolysis of 4-methylumbelliferyl-β-d-glucuronide (K_1_ = 1.32 ± 0.25 mmol/L, V_1_ = 1.2 mmol/mg/h) [23], while for the enzyme from the oxidoreductase group, the average value of the usual range for the Michaelis–Menten constant [24,25] was taken (K_2_ = 1 mmol/L), and the value for the maximum rate was arbitrarily determined (V_2_ = 1 mmol/mg/h).

Taking into account the mathematical model of the biosensor, at zero initial conditions, the transfer function of the biosensor was obtained:

(3) $$H\left(s\right)=\frac{P1\left(s\right)}{S\left(s\right)}=\frac{V_{1}\left(K_{2}s+V_{2}\right)}{K_{1}\left(K_{2}s+V_{2}\right)+K_{2}V_{2}}$$

From Fick’s and Faraday’s laws, for t > 0 and 0 < x < d, the mathematical model of the enzyme biosensor is obtained [22]:

(4) $$\frac{\partial S}{\partial t}=Ds\cdot \frac{\partial^{2}\cdot S}{\partial \cdot x^{2}}-\frac{V_{1}\cdot S}{K_{1}+S},$$

(5) $$\frac{\partial P1}{\partial t}=D_{p1}\frac{\partial^{2}P1}{\partial x^{2}}+\frac{V_{1}\cdot S}{K_{1}+S}+\frac{V_{2}\cdot P2}{K_{2}+P2},$$

(6) $$\frac{\partial P2}{\partial t}=D_{p2}\frac{\partial^{2}P2}{\partial x^{2}}-\frac{V_{2}\cdot P2}{K_{2}+P2},$$

where i is 1 or 2, t is time [s], x is space (length) [m], S(x,t) is the function concentration of the substance [mg/kg IBW], Pi(x,t) is the function of the concentration of the products P1 and P2 [mg/kg IBW], Vi(x,t) is the maximum rate of the enzyme reaction [mg/(kg IBW·s)], K_i_ is the Michaelis–Menten constant [mol/L], d is the thickness of the enzyme membrane [m] and, D_s_ and, D_p_ are the diffusion coefficients [m^2^/s].

It is assumed that x = 0 for the electrode surface and x = d for the solution-membrane interface; thus, the biosensor starts operating when the substrate appears on the enzyme membrane surface for t = 0:

S(x,0) = 0, 0 ≤ x < d,(7)

where S(d,0) = d0

Pi(x,0) = 0, 0 ≤ x ≤ d,(8)

where i = 1, 2.

The electrode potential is chosen to maintain a zero concentration of product P1 at the electrode surface. The rate of formation of product P2 at the electrode is proportional to the rate of conversion of product P1. When the substrate is homogeneously dispersed around the membrane, the diffusion layer remains of constant thickness (0 < x < d). Consequently, the concentration of substrate, as well as both products in the diffusion layer, remains constant while the biosensor comes into contact with the substrate solution. This property is used in the boundary conditions (t → ∞) given by:

(9) $$\frac{\partial S}{\partial x}{|}_{x=0}=0,S(d,t)=S0$$

(10) $$D_{p2}\frac{\partial P2}{\partial x}{|}_{x=0}=-D_{p1\;}\frac{\partial P1}{\partial x}{|}_{x=0}$$

where Pi(0,t) = 0 and, Pi(d,t) = 0.

The electric current of the biosensor depends on the flux P1 on the electrode surface for x = 0. Accordingly, the density iCEC (current density in the CEC scheme) at time t can be explicitly expressed from Faraday’s and Fick’s laws using the flux concentration P1 on the electrode surface:

(11) $$\mathrm{iCEC}(t)\;=\;n_{e}\;F\;D_{p1}\frac{\partial P1}{\partial x}{|}_{x=0}=-n_{e}\;FD_{p2}\frac{\partial P2}{\partial x}{|}_{x=0}$$

where n_e_ is the number of electrons involved in charge transfer at the electrode surface and, F is the Faraday’s constant. It is assumed that the system approaches a steady state for t → ∞:

(12) $$I_{\mathrm{cec}}\;=\;iCEC\left(t\right),$$

where I_cec_ is the steady state current of the biosensor.

### 2.3. The Pump

The infusion pump represented the actuator component responsible for LTG administration within the proposed control architectures. The pump received the control signal generated by the controller and delivered the corresponding LTG dose to the patient model. In the open-loop configuration, the control signal was determined from predefined therapeutic dosing parameters, whereas in the closed-loop configuration it was continuously adjusted according to the error between the target and measured LTG plasma concentrations. The objective of the closed-loop system was to minimize the concentration error and maintain LTG levels within the therapeutic range.

### 2.4. Mathematical Model of the Process and Controller Design

The pharmacokinetic process was described using a first-order linear model, in which the input represented the LTG administration rate and the output represented the LTG plasma concentration. Considering that lamotrigine pharmacokinetics can be approximated using a one-compartment model with first-order elimination kinetics [26,27,28], the patient-specific process model was defined as:

(13) $$\frac{dc_{i}}{dt}=-k_{e}c_{i}\left(t\right)+K_{i}\left(t\right)u_{i}\left(t\right)$$

where c_i_(t) is the LTG plasma concentration of the i-th patient, u_i_(t) is the LTG administration rate, k_e_ is the elimination rate constant, and K_i_ is the patient-specific process gain.

The elimination rate constant was calculated according to:

(14) ke=ln2t12

where t_1_/_2_ denotes the elimination half-life of lamotrigine [28]. The patient-specific process gain was estimated from the steady-state relationship between the administered LTG dose and the measured steady-state LTG plasma concentration reported in Table 1:

(15) $$K_{i}=\frac{k_{e}C_{{ss.}_{i}}}{{u\text{\_}c}_{{ss,}_{i}}}$$

where C_ss_,_i_ and u_ss,_i_ represent the steady-state LTG plasma concentration and the corresponding LTG dose for the i-th patient, respectively.

The corresponding transfer function of the process was given by:

(16) $$G_{i}\left(s\right)=\frac{C_{i}\left(s\right)}{U_{i}\left(s\right)}=\frac{K_{i}}{c+k_{e}}$$

A proportional–integral (PI) controller was selected due to its ability to reduce steady-state error while maintaining a relatively simple control structure suitable for biomedical drug delivery applications [21]. The control error was defined as:

(17) $$e\left(t\right)=r\left(t\right)-c\left(t\right)$$

where r(t) represents the reference LTG concentration and c(t) is the measured LTG plasma concentration obtained from the biosensor.

The PI controller output was calculated according to:

(18) $$u\left(t\right)=K_{p,REG}e\left(t\right)+K_{i,REG}\int e\left(t\right)dt$$

where K_p,REG_ and K_i,REG_ denote the proportional and integral gains, respectively. The control signal u(t) from Equation (18) determines the drug release rate from the implant. Therefore, this release rate is the only parameter that changes in real time based on biosensor measurements. Other delivery parameters, such as the duration of release events or the time intervals between them, remain unmodified during operation since the system relies on a continuous-release mechanism rather than a pulse-based delivery scheme. Furthermore, the controller parameters (K_p,REG_ and K_i,REG_) are not adjusted online. These patient-specific gains are calculated prior to treatment using the SIMC [29] tuning method to accommodate individual pharmacokinetic differences and remain fixed throughout the operating period.

### 2.5. Simulation of Intermittent Lamotrigine Release

**Figure 5:**
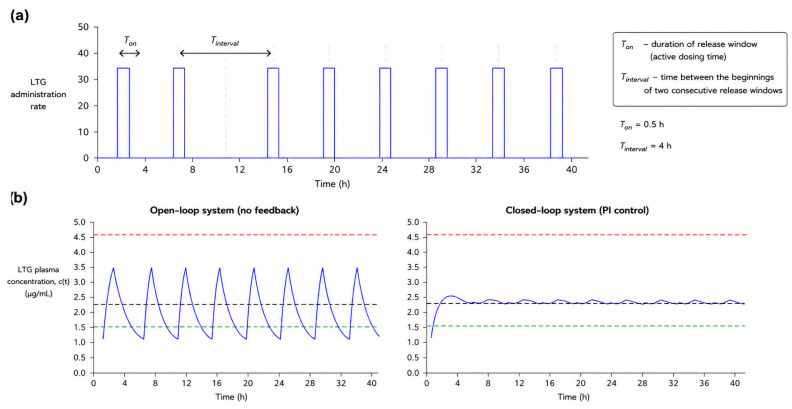
Simulation framework and simulated LTG plasma concentration responses of the proposed systems. Subfigures: (**a**) Intermittent LTG release profile used as the input signal for simulations; (**b**) Simulated LTG plasma concentration responses of the open-loop and closed-loop systems during intermittent LTG administration.

The simulation of intermittent LTG release from implants was performed using the Simulink graphical programming environment of MATLAB R2020b (MathWorks, Natick, MA, USA) on the Windows 10 operating system. The proposed simulation framework and the corresponding simulated responses of the open-loop and closed-loop systems are illustrated in Figure 5.

The intermittent LTG release profile used as the input signal in process is shown in Figure 5a. LTG administration was modeled as a sequence of predefined release intervals, where the infusion pump delivered the drug only during active release windows (T_on_), while the input signal was set to zero between two consecutive release intervals. The interval between the beginnings of two consecutive release windows was denoted as T_interval_. In the performed simulations, the duration of the active release window was set to T_on_ = 0.5 h, while the release interval was T_interval_ = 4 h.

The simulations were performed using the patient-specific pharmacokinetic process model described in Section 2.4 and the parameters derived from Table 1. The elimination rate constant was calculated from the LTG elimination half-life, while the process gain was estimated from the relationship between the administered LTG dose and the corresponding steady-state LTG plasma concentration.

Considering that approximately 91–92% of blood plasma consists of water [30] (ρ = 0.997 g/L, which can be approximated to 1 g/L), the plasma mass fraction (mg/kg) can be approximated as mass concentration (mg/L).

The simulation was performed using the values from Table 1. The molar LTG plasma concentrations were recalculated using the formula:

(19) $$c=\frac{\gamma \left(LTG\right)}{M\left(LTG\right)}$$

where c is the molar concentration [mmol/L], γ is the LTG mass concentration [mg/L] and M is the molar mass of lamotrigine, equal to 256.091 g/mol.

The plasmatic concentration used in the simulation varied between 0.002 and 0.058 mmol/L with mean value ± standard deviation equal to 0.018 ± 0.014 mmol/L for the observed patients. Adequately, the doses from Table 1 were transformed from mg/kg IBW in mmol/kg IBW. According to the literature, the commonly accepted therapeutic plasma concentration range for lamotrigine is approximately 3–15 μg/mL [31,32]. Therefore, the reference concentration used in the simulations was selected within this therapeutic interval and close to the mean steady-state concentration observed in Table 1 (4.67 μg/mL).

The simulated LTG plasma concentration responses for the open-loop and closed-loop systems are schematically presented in Figure 5b. In the open-loop configuration, LTG release was determined exclusively by the predefined dosing regimen without feedback correction, resulting in larger oscillations of LTG plasma concentration around the therapeutic range. In contrast, the closed-loop configuration continuously adjusted LTG administration using biosensor feedback and PI control, enabling more stable regulation of LTG plasma concentration around the reference value.

The systems were designed for chronic therapy, and all calculations were based on the assumption that the plasma lamotrigine concentration was already in the therapeutic range, i.e., more than five elimination half-life had passed from the first administration of the drug.

## 3. Results

**Figure 6:**
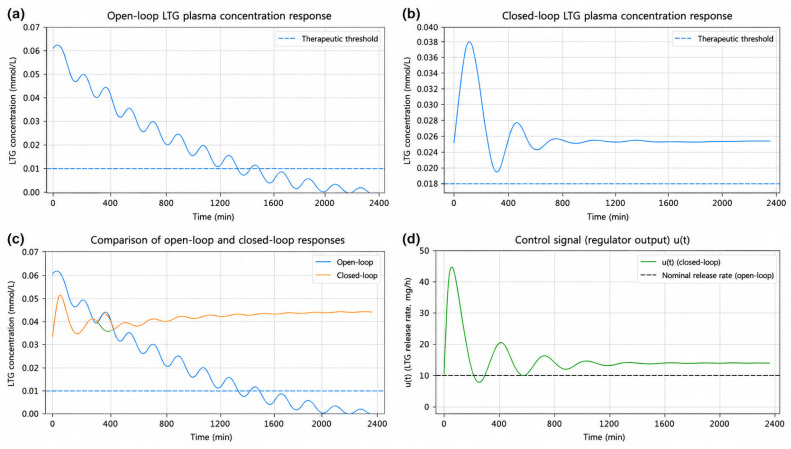
Simulation results of intermittent LTG delivery. Subfigures: (**a**) Simulated LTG plasma concentration response of the open-loop system with PI controller; (**b**) Simulated LTG plasma concentration response of the closed-loop system with PI control and biosensor feedback; (**c**) Comparison between the simulated open-loop and closed-loop responses; (**d**) Control signal generated by the PI controller. The dashed blue line indicates the therapeutic threshold concentration range (0.01 mmol/L).

The intermittent LTG release profile used as the input signal for the simulations is presented in Figure 6a. LTG administration was modeled as a sequence of periodic release intervals generated by the infusion pump. During each active release window, the pump delivered LTG according to the predefined dosing profile or the control signal generated by the controller, while no drug administration occurred between two consecutive release intervals.

The simulations were performed using the patient-specific pharmacokinetic process model and the biosensor model described in Section 2. The obtained simulation results enabled the evaluation of LTG plasma concentration dynamics during chronic intermittent therapy under both open-loop and closed-loop control configuration.

### 3.1. An Open-Loop Control System for LTG Delivery

The simulated response of the open-loop LTG delivery system is shown in Figure 6b. In this configuration, LTG administration was determined exclusively by the predefined dosing regimen without feedback correction based on the measured LTG plasma concentration.

The obtained results demonstrated pronounced oscillations of LTG plasma concentration during intermittent therapy. The simulated LTG concentration reached a maximum value of approximately 0.06 mmol/L approximately 6 min after drug administration. However, due to the absence of feedback regulation, the concentration continuously decreased and fell below the therapeutic concentration threshold of 0.01 mmol/L after approximately 1600 min.

Although the open-loop system successfully generated intermittent LTG delivery, the obtained response indicated limited capability for maintaining stable therapeutic concentrations over prolonged periods. Larger concentration fluctuations and increased sensitivity to dosing intervals were observed throughout the simulation.

### 3.2. Closed-Loop System for LTG Delivery

The simulated response of the closed-loop LTG delivery system with biosensor-based feedback and PI control is presented in Figure 6c. In this configuration, the biosensor continuously monitored LTG plasma concentration and provided feedback information to the controller, which dynamically adjusted the administered LTG dose.

Compared with the open-loop configuration, the closed-loop system demonstrated substantially improved regulation of LTG plasma concentration. The simulated concentration reached a maximum value of approximately 0.045 mmol/L approximately 6 min after LTG administration and subsequently stabilized within the therapeutic interval of 0.02–0.03 mmol/L.

The PI controller successfully compensated for concentration deviations introduced by intermittent LTG release, resulting in reduced concentration oscillations and improved maintenance of the desired therapeutic concentration range. No significant concentration decrease below the therapeutic threshold was observed during the simulated interval.

### 3.3. Comparison of Open-Loop and Closed-Loop Configurations

A comparison between the simulated open-loop and closed-loop responses is shown in Figure 6d and summarized in Table 2. The obtained results demonstrated that the inclusion of biosensor-based feedback significantly improved the regulation of LTG plasma concentration during intermittent therapy. In the open-loop system, the absence of feedback correction resulted in larger concentration oscillations and faster decline of LTG concentration below the therapeutic range. In contrast, the closed-loop configuration enabled more stable concentration dynamics and improved maintenance of therapeutic LTG levels through continuous adjustment of drug administration.

These results indicate that the proposed closed-loop architecture may provide more reliable long-term LTG concentration regulation and could potentially improve the safety and efficiency of chronic LTG therapy.

## 4. Discussion

Although the first data on epilepsy date back to ~1000 BC, there are still many difficulties in the treatment of both diseases [6]. The goal of pharmacotherapy of epilepsy is to achieve the complete absence of epileptic seizures, while reducing morbidity and mortality, and without causing serious side effects, which together contribute to improving the quality of life of the affected person [33]. However, the occurrence of serious side effects is often a factor that determines the discontinuation of therapy, and in the context of LTG, use is discontinued due to the occurrence of a hypersensitivity reaction, which manifests itself as a rash or impaired liver function [34]. In a significant number of patients, the use of this active principle is associated with sleep disturbances [35], dizziness, diplopia, nausea and tremor [7]. Although acute LTG overdose is benign in most cases, rare potentially life-threatening conditions have been reported, such as epileptic seizures, coma, hypotension, tachycardia and cardiac arrest [36].

Studies suggest that the most common reason for discontinuation of therapy is insufficient potency of the administered antiepileptic drugs [37], which may be due to low maximum daily doses of the drug [38]. LTG dosing is individualized for both loading and maintenance doses [7], while recommendations are to maintain plasma concentrations in the range of 0.01–0.05 mmol/L during chronic therapy [19]. In accordance with this therapeutic range, an implant in an open-loop system was designed that delivers a precisely determined dose of LTG in a defined time interval (*ττ*).

Simulations have shown that after delivery of a single random dose, plasma Cmax is achieved that exceeds the recommended therapeutic range (0.06 mmol/L vs. 0.05 mmol/L), which increases the likelihood of adverse reactions [19]. After subcutaneous absorption of delivered LTG, Cmax is achieved after approximately 6 min, while after intestinal absorption, Cmax is reached in 2.8 ± 1.3 h [15]. The simulation predicts that plasma LTG concentrations will fall below the therapeutic range as early as 1600 min after dose delivery. It should be noted that the open-loop response in Figure 5b represents a carefully pre-calculated intermittent dosing regimen where the intervals and drug amounts are optimized in advance. In contrast, Figure 6b demonstrates the baseline response when the controller operates without feedback error information, resulting in a constant control signal u(t) and providing a direct basis for comparison with the closed-loop system.

On the other hand, a closed-loop system not only delivers LTG but also provides feedback on plasma levels. The first enzyme in the biosensor, β-glucuronidase, catalyzes the hydrolysis of LTG-N-2-glucuronide, which is the dominant metabolite of LTG in the systemic circulation (76% of total metabolites [39]), ensuring the reward of the enzyme-metabolite complex. Another enzyme from the oxidoreductase group performs the cyclic conversion of LTG from the oxidized to the reduced form and vice versa, which generates an electrochemical potential on the surface of the biosensor that is detected. The resulting signal is directly proportional to the level of LTG-N-2-glucuronide in plasma. By processing this signal, the system recognizes the required dose of LTG and delivers it, and after 6 min a concentration maximum (Cmax = 0.045 mmol/L) is achieved, which is within the therapeutic range (0.045 mmol/L vs. 0.05 mmol/L). Plasma levels of LTG are maintained within the therapeutic range, reaching a plateau at values of 0.02–0.03 mmol/L.

An open-loop system requires fewer components to implement and is therefore cheaper and simpler, unlike a closed-loop system, which includes a biosensor in addition to the existing components of the first system. Accordingly, modeling is more practical, as is the mathematical tool used to implement an open-loop system. Another advantage is that the stability of the process cannot be compromised, unlike a closed-loop system. However, a key drawback of open-loop systems is the lack of feedback on the delivery of a given LTG concentration, which is a problem if a disturbance occurs in the system, such as the interaction of LTG with another substance [21].

In practice, the closed-loop system is almost exclusively used because it is resistant to modeling errors and external disturbances, provides feedback on the state of the system, and thus allows for more efficient control and error correction, which has proven to be a necessity in the context of intermittent LTG dosing [21].

Several challenges may arise when translating the platform proposed in this study from the design stage to real-world applications. As the proposed biosensor system has not yet been experimentally realized, analytical performance metrics such as the limit of detection, linear range, calibration characteristics, selectivity, and long-term stability remain to be established. Future studies will focus on experimental validation of the platform, including interference testing against co-administered antiepileptic drugs, endogenous biomolecules, and major LTG metabolites to assess its suitability for therapeutic drug monitoring in clinical settings. Another limitation of the current model is the assumption of direct LTG entry into the systemic circulation. In practice, subcutaneous drug delivery may be affected by local transport barriers, including tissue perfusion, membrane diffusion, capillary permeability, fibrous encapsulation, extracellular matrix density, and interstitial fluid pressure [40,41]. These factors may introduce delays and variability in drug absorption and should be evaluated in future experimental studies. Moreover, surrogate kinetic parameters for β-glucuronidase and estimated values for the oxidoreductase reaction were applied in the design. These assumptions were adopted due to the lack of experimentally determined kinetic data for the specific enzyme–substrate systems considered. Consequently, the model should be viewed as a proof-of-concept framework intended to illustrate system behavior rather than provide quantitative predictions. Future experimental studies will be required to determine substrate-specific kinetic constants and validate the model under physiologically relevant conditions.

Since an enzyme-coupled biosensor is used, the instability of the enzyme, i.e., its potential denaturation under the influence of temperature, pH or some chemical agent, will be reflected in the instability of the entire system. Enzyme denaturation and biofouling represent the two principal mechanisms underlying the failure of enzyme-based biosensors. Biofouling impedes analyte transport to the sensor surface through the accumulation of unwanted biological materials, whereas enzyme denaturation results in the loss of the enzyme’s native conformation and catalytic activity. Moreover, biofouling can exacerbate enzyme degradation by creating a hostile microenvironment at the sensor interface, thereby accelerating the decline in sensor sensitivity, operational stability, and overall lifespan [42]. To mitigate biofouling and enzyme degradation, various material and fabrication strategies can be tested in subsequent investigations to ensure the stability of the biosensor. Some of the possible strategies include: (i) anti-fouling coatings based on hydrophilic, zwitterionic, or poly(ethylene glycol) polymers, (ii) advanced immobilization techniques, such as covalent attachment and entrapment within hybrid silica matrices or conductive hydrogels, and (iii) enzyme encapsulation in protective nanostructures and the use of nanozymes [43,44,45,46].

This work represents an interdisciplinary approach to developing a modern therapeutic system, in which drug delivery would function independently of the dynamics of the patient’s life, reducing the risk of inadequate oral drug administration. A proposal for the design of the implant itself is given, as well as initial in silico confirmation of its functioning, but further confirmation of its effectiveness requires the construction of the implant and its implementation in vivo, as well as clinical studies.

## 5. Conclusions

Multiple etiologies, a wide range of adverse reactions, the need for combination therapy, interindividual differences, the difficulty of achieving and maintaining therapeutic doses, as well as the many interactions of antiepileptic drugs, are just some of the reasons why it is necessary to consider an alternative, modern approach to therapy. The open-loop system automatically delivers a given dose of LTG and does not provide information about the drug used. Simulation of this system has shown that plasma concentrations of LTG after oral administration are maintained in the therapeutic range for a longer period, compared to the dose delivered from the implant. In addition to reduced efficacy, implant placement is an invasive procedure, and its use in an open-loop system is not justified. The closed-loop system estimates the required dose based on monitoring of plasma LTG concentrations and it is capable to deliver the required dose. The simulation results showed that the use of this system successfully maintains plasma LTG concentrations within the therapeutic range. Its application has the potential to eliminate human error factors in dosing, such as incorrect medication administration, subdose or overdose, skipping a dose, or taking the drug at the wrong time. The development of an implant that intermittently releases lamotrigine with simultaneous monitoring could significantly improve the effectiveness of epilepsy therapy and improve the quality of life of patients.

## Figures and Tables

**Table 2:** Comparison of simulated open-loop and closed-loop LTG delivery systems.

Parameter	Open-Loop	Closed-Loop
Maximum LTG concentration [mmol/L]	0.06	0.045
Time to peak concentration [min]	6.0	6.0
Stable concentration range [mmol/L]	Not achieved	0.02–0.03
Feedback correction	No	Yes
Concentration below therapeutic threshold	After 80 min	Not observed

## Data Availability

The datasets used and analyzed in the current study are available from the corresponding author on reasonable request.

## Abbreviations

The following abbreviations are used in this manuscript:

LTG	lamotrigine
ILAE	International League Against Epilepsy
WHO	World Health Organization
OCT	Organic cationic transporter
IBW	Ideal body weight
BW	body weight
H	height
PI	proportional–integral
